# High urinary excretion of kidney injury molecule-1 predicts adverse outcomes in acute kidney injury: a case control study

**DOI:** 10.1186/s13054-016-1455-6

**Published:** 2016-09-10

**Authors:** Yuanyuan Xie, Qin Wang, Chunlin Wang, Chaojun Qi, Zhaohui Ni, Shan Mou

**Affiliations:** 1 Department of Nephrology, Molecular Cell Laboratory for Kidney Disease, Renji Hospital, School of Medicine, Shanghai Jiaotong University, Shanghai, People’s Republic of China; 2 Shanghai Center for Peritoneal Dialysis Research, Shanghai, People’s Republic of China

**Keywords:** Acute kidney injury, Urine kidney injury molecule-1, Renal AKI, Transient AKI, Long-term outcomes

## Abstract

**Background:**

Acute kidney injury (AKI) is a common clinical syndrome with poor prognosis. The insensitivity and non-specificity of traditional markers of renal dysfunction prevent timely estimation of the severity of renal injury, and the administration of possible therapeutic agents. Urinary kidney injury molecule-1 (uKIM-1) is a marker of epithelial injury of renal tubules. Different uKIM-1 levels are associated with various degrees of renal injury. This study sought to evaluate uKIM-1 as a predictor of renal prognosis by analyzing uKIM-1 levels in patients with AKI.

**Methods:**

A total of 258 patients were screened, 201 patients were enrolled in the study, and 17 patients were lost to follow up. Therefore, 184 AKI patients were included in this study and were classified into transient AKI and renal AKI groups according to short-term renal function recovery (48 h). Changes in renal function were observed for one year during regular follow up, and risk factors that affected renal prognosis were analyzed.

**Results:**

The uKIM-1 level in the renal AKI group was significantly higher than that in the transient AKI group. The receiver operating characteristic area under the curve (ROC-AUC) of uKIM-1 for the diagnosis of renal AKI was 0.691, and its sensitivity and specificity were 66.3 % and 64.7 %, respectively. The uKIM-1 level at AKI occurrence was significantly higher in the group with deterioration in renal function than in the group with stable renal function. Thus, uKIM-1 level is a prognostic factor for poor renal prognosis. ROC curve analysis demonstrated that the AUC for the prediction of renal function progression on the basis of uKIM-1 levels in patients with renal AKI and AKI was 0.680 and 0.703, respectively; the sensitivity was 78.6 % and 78.4 %, respectively; and the specificity was 57.9 % and 60.8 %, respectively. uKIM-1 > 2.37 ng/mg in patients with AKI positively correlated with poor renal prognosis.

**Conclusions:**

uKIM-1 levels sensitively predict the renal prognosis of patients with AKI, and they may be used as early screening indicators for poor renal prognosis.

## Background

Acute kidney injury (AKI) is one of a number of conditions that affect kidney structure and function. AKI is defined by an abrupt decrease in kidney function. It is a broad clinical syndrome encompassing various types of etiology [1]. AKI has a high incidence rate, and it is an increasingly common complication associated with poor prognosis in hospitalized patients. AKI consumes a large amount of social and medical resources, but there is a shortage of effective therapeutic regimens [2, 3]. The kidney normally exhibits an extremely strong capacity for self-regeneration and repair, and can recover from mild and moderate injury [4]. However, incomplete renal repair can result in chronic kidney disease (CKD) in some cases [4, 5]. In recent years, the incidence rate of CKD has annually increased with the increasing incidence rate of AKI [6]. Numerous studies have found that renal function in some patients with AKI does not recover completely but gradually progresses to CKD and end-stage renal disease (ESRD), and may require permanent renal replacement therapy [2, 6, 7]. Urinary kidney injury molecule-1 (uKIM-1) is a marker of epithelial injury of renal tubules, and it is elevated in the early stages of AKI [8]. Different uKIM-1 levels are associated with various degrees of renal injury. This study examined the value of uKIM-1 level in identifying reversible renal injury and its predictive value for long-term prognosis by examining renal function recovery during short-term and long-term prognoses in patients with AKI.

## Methods

### Study subjects

Male and female inpatients ≥18 years of age with complete clinical data who were diagnosed with AKI or acute-on-chronic kidney injury (A on C) were enrolled in this study. Minor patients and patients with a life expectancy less than one year because of malignant disease were excluded. AKI was defined and staged using the Kidney Disease Improving Global Outcomes (KDIGO) Clinical Practice Guidelines for Acute Kidney Injury [1].

Our study was conducted at Renji Hospital, School of Medicine, Shanghai Jiaotong University, Shanghai, P.R. China, a 2000-bed teaching hospital. The subjects were recruited from all hospital departments (both surgical and non-surgical), including ICU.

### Sampling

Fresh urine and blood samples were obtained from each patient at the time of AKI diagnosis and at 24 h and 48 h, and at the 3-month, 6-month, and 12-month follow up, and they were submitted to a biochemical laboratory for analysis. Fresh urine (10 ml) collected from each patient at the time of AKI diagnosis was centrifuged at 1000 × g for 15 minutes, and the liquid supernatant was placed into Eppendorf tubes and stored at -80 °C until subsequent analysis.

### Detection of indicators

Serum creatinine (sCr) and urine creatinine (uCr) were detected using enzymatic methods. A simplified formula for the modification of diet in renal disease (MDRD) was used to calculate the estimated glomerular filtration rate (eGFR):

eGFR = 186 × (sCr/88.4)^‐ 1.154^ × Age^‐ 0.203^ × (0.742, Female) [9].

An enzyme-linked immunosorbent assay (ELISA) was performed to detect urine kidney injury molecule-1 (uKIM-1) using a kit from the American R&D Corporation (Minneapolis, MN, USA). The concentration of the tested sample was calculated using a standard curve, and the results are expressed in ng/mg after synchronous uCr correction. Fractional excretion of sodium (FeNa) was calculated as follows:

$$ \mathrm{FeNa} = \left(\mathrm{Plasma}\ \mathrm{creatinine} \times \mathrm{Urinary}\ \mathrm{N}\mathrm{a}\right)/\left(\mathrm{Plasma}\ \mathrm{N}\mathrm{a} \times \mathrm{Urinary}\ \mathrm{creatinine}\right). $$

The renal failure index (RFI) was calculated as follows:

$$ \mathrm{R}\mathrm{F}\mathrm{I}=\left(\mathrm{Urinary}\ \mathrm{N}\mathrm{a} \times \mathrm{Plasma}\ \mathrm{creatinine}\right)/\mathrm{Urinary}\ \mathrm{creatinine}. $$

### Grouping

Patients with AKI were divided into transient AKI and renal AKI groups according to changes in renal function within 48 h. Transient AKI was defined as a reversal within 48 h [10]. Renal AKI was defined as non-transient AKI when no recovery was observed within 48 h [10]. Recovery of AKI was determined as reduction of serum creatinine from the peak to less than 0.3 mg/dl above baseline [10]. We also corrected sCr for fluid balance [11]. No patients received blood transfusions.

Patients with AKI and patients with renal AKI patients were classified into two groups during one year of follow up: a group with stable renal function and a group with deterioration in renal function. Deterioration in renal function was defined as a one-stage reduction in GFR accompanied by a 25 % or greater reduction in eGFR from baseline [12].

### Statistical analysis

SPSS13.0 statistical software IBM (NY, USA) was used. Normally distributed measurement data are expressed as mean ± sd, and the *t* test was used for group comparisons. Measurement data with a non-normal distribution are expressed as the median (M) and interquartile range (P25, P75). The rank sum test was used for comparisons between groups. Spearman correlation analysis was used to analyze correlations. A receiver operating characteristic (ROC) curve and the area under curve were used to calculate the sensitivity and specificity. Logistic regression analysis was used to analyze related risk factors that influenced the prognosis of kidney function. Cox multifactor regression analysis was used to analyze the relationship between all risk factors and the life span of the kidneys. Survival rate was analyzed using a Kaplan-Meier life survival curve. A difference of *P* < 0.05 was considered statistically significant.

## Results

### General data

**Fig. 1:**
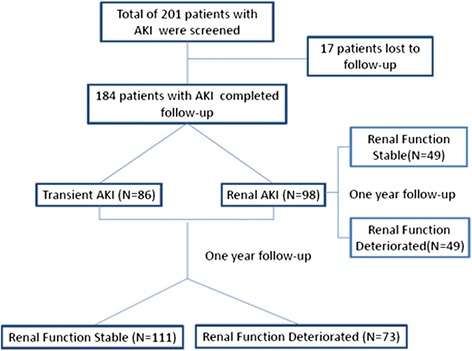
Study flow chart. *AKI* acute kidney injury

A total of 258 patients were screened, 201 patients were enrolled in the study, and 17 patients were lost to follow up. Therefore, 184 patients with AKI (86 patients with transient AKI and 98 patients with renal AKI) were included in the study. All the 184 patients survived to one-year follow up. After regular follow up for one year, 111 of the 184 patients were in the stable renal function group, and 73 patients were in the renal function deterioration group. Of the 98 patients with renal AKI, 49 were in the stable renal function group, and 49 were in the renal function deterioration group (Fig. 1).

Of the 184 patients with AKI, the ages ranged from 18 to 99 years, and the median age was 53.00 (31.00, 64.75) years. The male/female ratio was 1.75:1, and the mean follow-up duration was 12.2 ± 1.06 months. The basic serum creatinine (sCr), basic eGFR and basic 24-h total urine protein levels were 1.12 (0.87, 2.40) mg/dl, 66.68 (28.10, 94.18) ml/min/1.73 m^2^ and 3.78 (1.47, 9.08) g, respectively. The baseline creatinine was considered the lowest value within 3 months prior to diagnosis of AKI.

There were 71 patients with previous chronic renal disease (CKD). CKD is defined according to Kidney Disease Improving Global Outcomes (KDIGO) Clinical Practice Guidelines for the Evaluation and Management of Chronic Kidney Disease [12]. The uKIM-1 level at the time of AKI occurrence was 2.37 (1.10, 6.22) ng/mg, and the peak level of creatinine was 2.43 (1.41, 3.74) mg/dl. There were 123 patients in AKI stage I, 26 patients in stage II and 35 patients in stage III. Patients with renal AKI accounted for 53.26 %. The clinical etiology of AKI included insufficient renal blood perfusion or ischemia (22 %) (insufficient volume, gastrointestinal loss, cardiac failure, or renal vascular factors), nephrotoxicity (20 %) (nephrotoxic agents and contrast medium injury, etc.), infection factor (23 %) (sepsis, severe pneumonia and any kind of infection that caused renal injury), aggravation or activation of glomerular disease (19 %), and obstruction (11 %). The proportion of patients with renal function progression was 39.67 % at the one-year follow up (Table 1).

**Table 1:** General condition of patients

Characteristics	Values (*n* = 184 patients)
Age, years	53.00 (31.00, 64.75)
Male sex (*n*, %)	117 (63.59 %)
Oliguria (*n*, %)	9 (4.9 %)
Baseline serum creatinine (mg/dl)	1.12 (0.87, 2.40)
Baseline estimated GFR (ml/min/1.73 m^2^)	66.68 (28.10, 94.18)
Baseline 24 hUTP (g/24 h)	3.78 (1.47, 9.08)
sAlb (g/l)	33.30 (24.00, 40.25)
Hb (g/l)	119.00 (99.25, 140.00)
ALT (IU/l)	14.00 (10.00, 23.00)
AST (U/l)	17.00 (14.00, 25.75)
Ca (mmol/l)	2.08 (1.99, 2.22)
P (mmol/l)	1.32 (1.10, 1.56)
K (mmol/l)	3.90 (3.40, 4.30)
HCO_3_^-^(mmol/l)	24.20 (20.90, 26.40)
hsCRP (mg/l)	2.40 (0.80, 8.17)
Cause of AKI (*n* (%)	
Ischemia	40 (21.74 %)
Nephrotoxicity	37 (20.11 %)
Infection	42 (22.83 %)
Glomerular disease	35 (19.02 %)
Obstruction	21 (11.41 %)
Others	9 (4.90 %)
AKI stage, *n*	
Stage 1	123
Stage 2	26
Stage 3	35
Peak serum creatinine (mg/dl)	2.43 (1.41, 3.74)
uKIM-1 (ng/mg)	2.37 (1.10, 6.22)
FeNa ≥1 % (*n* (%))	126 (68.48 %)
RFI ≥1 % (*n* (%))	136 (73.91 %)
Renal AKI (*n* (%))	98 (53.26 %)
Progression of renal failure (*n* (%))	73 (39.67 %)

Conversion factor for serum creatinine in mg/dL to mmol/L, ×88.4. *AKI* acute kidney injury, *GFR* glomerular filtration rate, *UTP* urinary protein excretion, *sAlb* serum albumin, *Hb* hemoglobin, *ALT* alanine aminotransferase, *AST* aspartate aminotransferase, *Ca* calcium, *P* phosphorus, *K* potassium, HCO_3_^-^, bicarbonate, *hsCRP* high-sensitivity C-reactive protein, *uKIM-1* urinary kidney injury molecule-1, *FeNa* fractional excretion of sodium, *RFI* renal failure index

### uKIM-1 detection by western blot

**Fig. 2:**
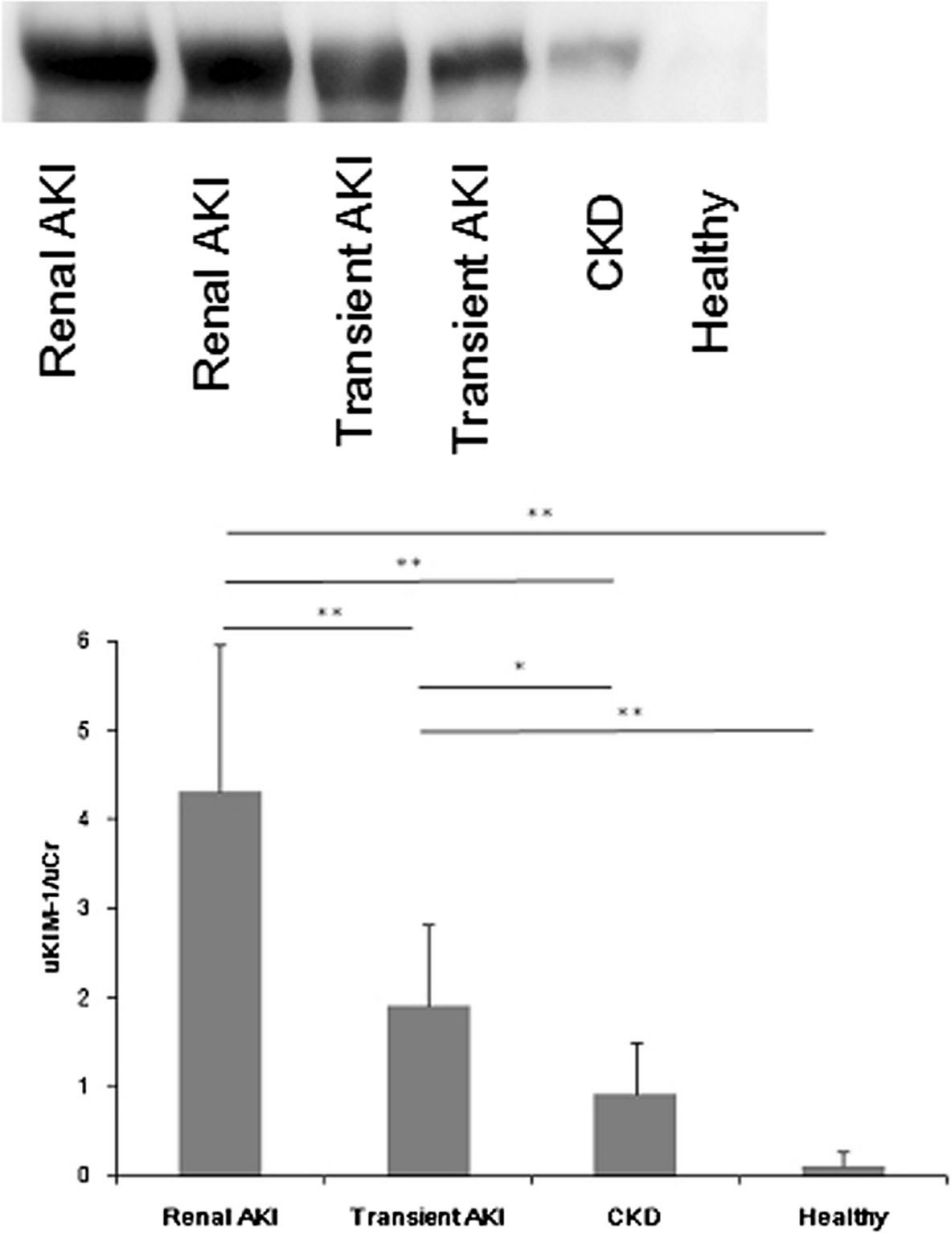
Western blot analysis of urinary kidney injury molecule-1 (*uKIM-1*) with and without established acute kidney injury (*AKI*). All patients with established AKI had easily detectable uKIM-1. The uKIM-1 levels in urine from patients who were clinically diagnosed with renal AKI were higher than in patients with transient AKI: *P < 0.05, **P < 0.001. *CKD* chronic kidney disease

Western blot analysis was performed on urine specimens from six patients: two patients were clinically diagnosed with renal AKI, two patients were clinically diagnosed with transient AKI, one was a patient with CKD, and one patient was used as a normal control. uKIM-1 was undetectable in healthy control urine, and a low level was observed in the urine of the patient with CKD. In sharp contrast, all patients with established AKI had easily detectable levels of uKIM-1, and higher levels were observed in urine from patients who were clinically diagnosed with renal AKI (Fig. 2).

### Correlation analysis

**Fig. 3:**
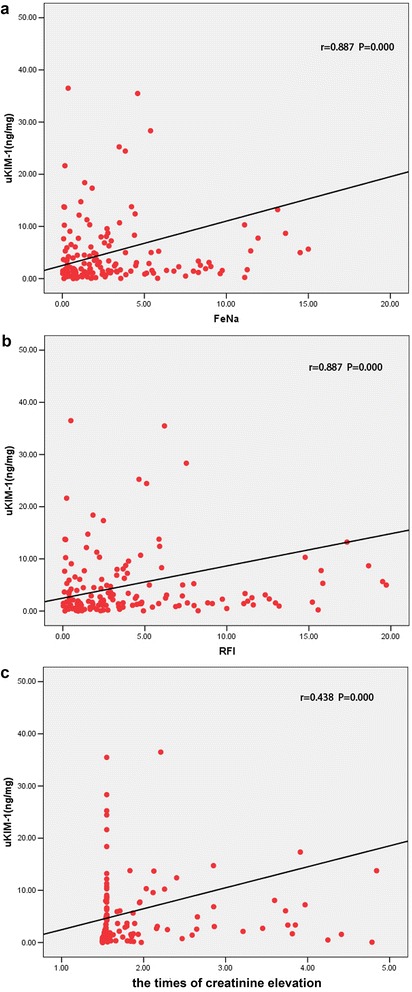
Spearman correlation analyses. **a** The urinary kidney injury molecule-1 (*uKIM-1*) content was positively correlated with fractional excretion of sodium (*FeNa*). **b**. The uKIM-1 content was positively correlated with the renal failure index (*RFI*). **c**. The uKIM-1 content was positively correlated with the times of creatinine elevation

Spearman correlation analysis indicated that uKIM-1 positively correlated with the fractional excretion of sodium (FeNa), renal failure index (RFI) and creatinine elevation (*r* = 0.887, 0.887 and 0.438, respectively, *P* = 0.000) (Fig. 3).

### Identification of transient AKI and renal AKI on the basis of uKIM-1 levels

**Fig. 4:**
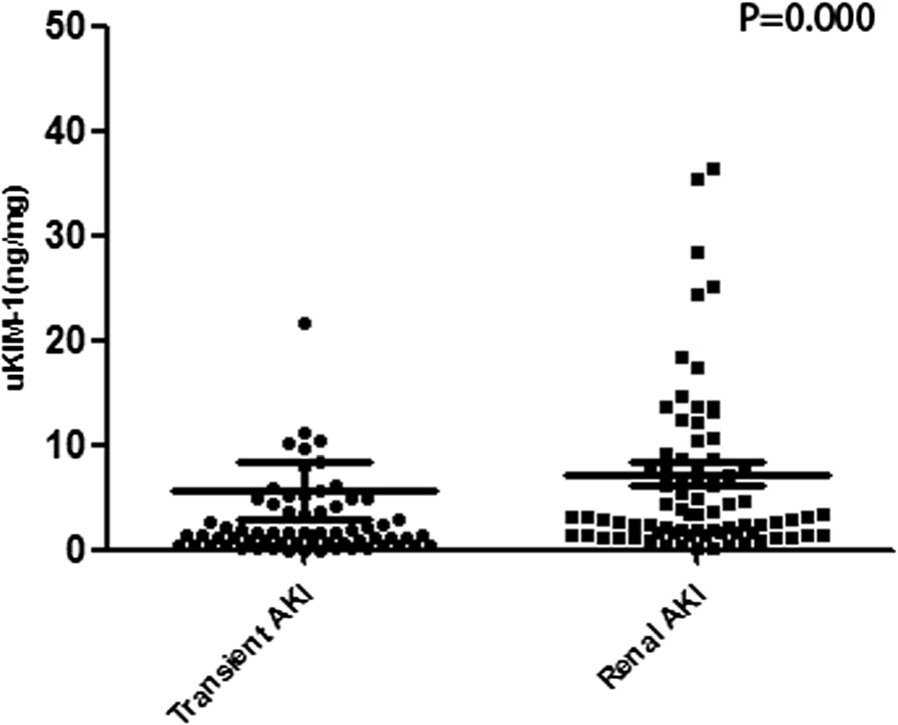
The urinary kidney injury molecule-1 (*uKIM-1*) levels in the transient acute kidney injury (*AKI*) group were significantly higher than those of the renal AKI group

The patients were classified into transient AKI (86 patients) and renal AKI groups (98 patients) on the basis of renal function recovery during short-term follow up (≤48 h). Comparisons between groups demonstrated that the prognosis of the renal AKI group was significantly inferior to the transient AKI group. Of the 86 patients with transient AKI, 44 presented with a FeNa ≥1 %, and 48 presented with an RFI ≥1 %. Comparisons between groups indicated that the uKIM-1 level of the renal AKI group was significantly higher than that of the transient AKI group (Table 2, Fig. 4).

**Table 2:** Comparison between the transient AKI group and the renal AKI group

	Transient AKI (*N* = 86)	Renal AKI (*N* = 98)	*P*
uKIM-1 (ng/mg)	1.52 (0.57, 4.29)	3.24 (1.56, 8.53)	0.000
FeNa ≥1 % (*n* (%))	44 (51.16 %)	82 (83.67 %)	0.000
RFI ≥1 % (*n* (%))	48 (55.81 %)	88 (89.80 %)	0.000
Progression of renal failure (*n* (%))	24 (27.91 %)	49 (50.00 %)	0.000

*AKI* acute kidney injury, *uKIM-1* urinary kidney injury molecule-1, *FeNa* fractional excretion of sodium, *RFI* renal failure index

**Fig. 5:**
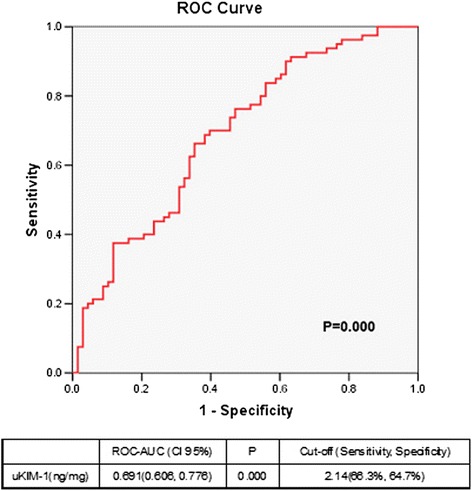
Receiver operating characteristic area under the curve (*ROC-AUC*) of renal acute kidney injury according to urinary kidney injury molecule-1 (*uKIM-1*) level

ROC curve analysis revealed that the area under the curve (ROC-AUC) was 0.691 (*P* = 0.000) for the diagnosis of renal AKI on the basis of uKIM-1. The sensitivity was 66.3 % when the cutoff point of uKIM-1 level was 2.14 ng/mg, and the specificity was 64.7 % (Fig. 5).

### Analysis of long-term prognosis predictions on the basis of uKIM-1 levels

#### Analysis of long-term prognosis for patients with renal AKI

Of the 98 patients with renal AKI at the regular one-year follow up, 49 were in the stable renal function group, and 49 were in the renal function deterioration group. There was no difference by sex in the composition of the groups. However, patients in the renal function deterioration group were older than those in the renal function stable group, and their basic renal function was inferior, with a high peak value of creatinine after AKI. More patients in the renal function deterioration group presented with FeNa ≥1 % and RFI ≥1 %, and their uKIM-1 levels were significantly higher than the stable renal function group (Table 3).

**Table 3:** Comparison between the renal function deterioration group and the stable renal function group after one-year follow up in patients with renal acute kidney injury

Characteristics	Stable renal function (*n* = 49)	Renal function deterioration (*n* = 49)	*P*
Age, years	53.00 (32.75, 65.00)	62.00 (45.00, 72.00)	0.039
Male sex, (*n* (%))	30 (61.22 %)	31 (63.27 %)	1.000
Baseline serum creatinine (mg/dl)	1.12 (0.87, 1.88)	2.23 (1.11, 3.13)	0.006
Baseline estimated GFR (ml/min/1.73 m^2^)	74.00 (39.31, 92.33)	30.52 (17.01, 53.93)	0.001
Baseline 24 hUTP (g/24 h)	6.12 (2.13, 9.34)	5.32 (1.58, 9.48)	0.563
Peak serum creatinine (mg/dl)	2.20 (1.49, 3.63)	3.43 (2.44, 4.51)	0.002
uKIM-1 (ng/mg)	2.05 (1.17, 6.33)	5.15 (2.48, 11.06)	0.006
FeNa ≥1 % (*n* (%))	26 (53.06 %)	44 (89.80 %)	0.000
RFI ≥1 % (*n* (%))	32 (65.31 %)	47 (95.92 %)	0.000

Conversion factor for serum creatinine in mg/dL to mmol/L, ×88.4. *GFR* glomerular filtration rate, *UTP* urinary protein excretion, *uKIM-1* urinary kidney injury molecule-1, *FeNa* fractional excretion of sodium, *RFI* renal failure index

Gradual Cox regression analysis indicated that patients with renal AKI had higher uKIM-1 levels with poorer long-term renal prognosis. The risk of renal function deterioration following AKI increased by 6.4 % for each 1 ng/mg increase in uKIM-1. The prognosis of patients with renal AKI who presented with FeNa ≥1 % was inferior to that of patients with FeNa <1 % (Table 4).

**Table 4:** Cox regression shows the independent risk factors for renal function progression in patients with renal acute kidney injury

	Univariate analysis		Multivariate analysis	
Characteristics	Relative risk	*P*	Relative risk	*P*
Age, years	1.025 (1.006, 1.044)	0.009		
Male sex				
Baseline estimated GFR (ml/min/1.73 m^2^)	0.974 (0.959, 0.990)	0.001		
Peak serum creatinine (mg/dl)	1.002 (1.000, 1.003)	0.009		
uKIM-1 (ng/mg)	1.064 (1.036, 1.093)	0.000	1.064 (1.024, 1.106)	0.001
FeNa ≥1 %	4.510 (1.588, 12.806)	0.005	9.538 (1.264, 72.006)	0.029
RFI ≥1 %	5.486 (1.311, 22.945)	0.020		

*GFR* glomerular filtration rate, *uKIM-1* urinary kidney injury molecule-1, *FeNa* fractional excretion of sodium, *RFI* renal failure index

**Fig. 6:**
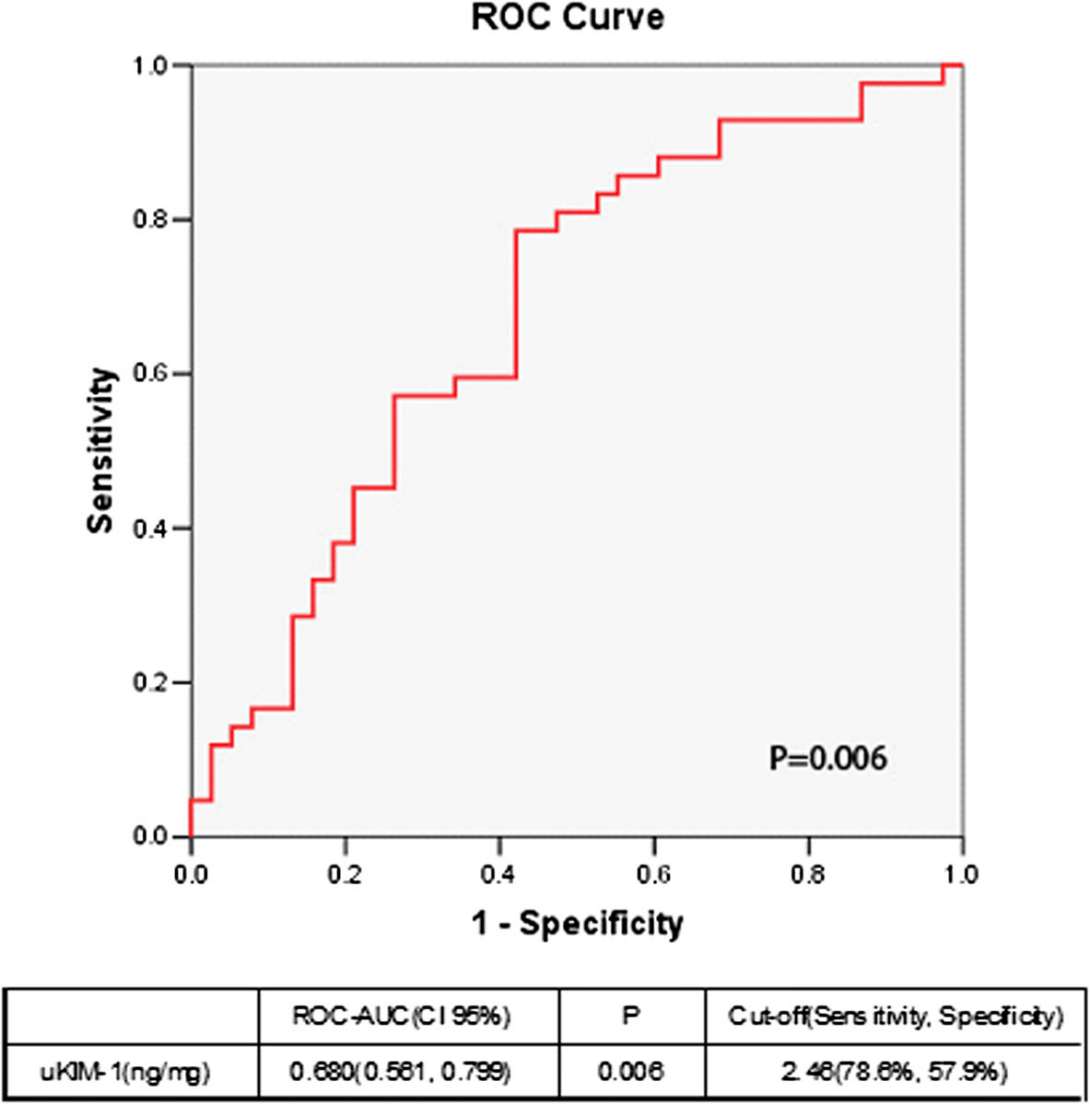
Receiver operating characteristic area under the curve (*ROC-AUC*) of renal function progression in patients with renal acute kidney injury

**Fig. 7:**
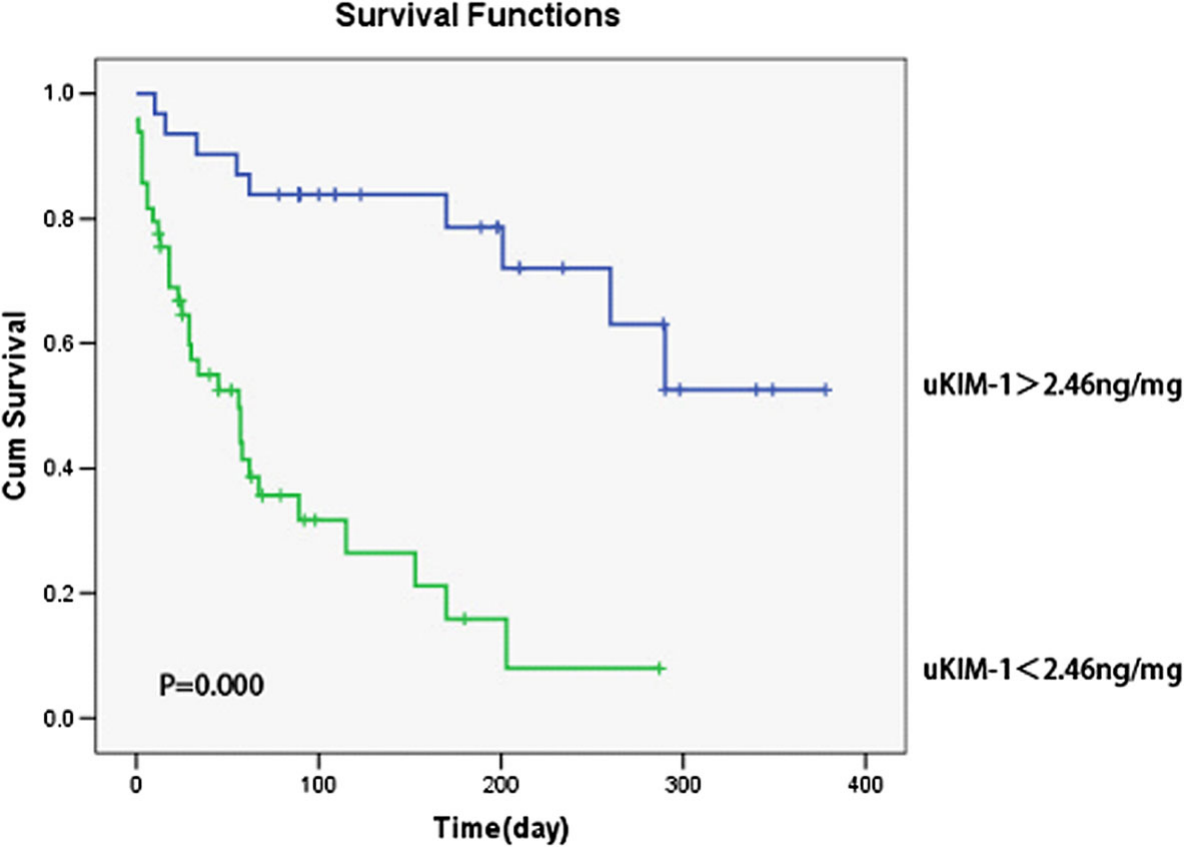
Urinary kidney injury molecule-1 (*uKIM-1*) level and kidney prognosis in patients with renal acute kidney injury patients. uKIM-1 > 2.46 ng/mg was positively related to poor prognosis. *Cum* cumulative

The ROC-AUC for the prediction of renal function deterioration in patients with renal AKI when the uKIM-1 level was 0.680. When the cutoff for uKIM-1 was 2.46 ng/mg, the sensitivity was 78.6 %, and the specificity 57.9 % (Fig. 6). The K-M curve indicated that uKIM-1 > 2.46 ng/mg positively correlated with poor long-term renal prognosis in patients with renal AKI (*P* = 0.000) (Fig. 7).

#### Analysis of long-term prognosis for patients with AKI

Of the 184 patients with AKI after the regular one-year follow up, 111 patients were in the stable renal function group, and 73 patients were in the renal function deterioration group. There was no difference by sex in the composition of the groups. However, patients in the renal function deterioration group were older than patients in the stable renal function group, and their basic renal function was inferior, with a high peak value of creatinine following AKI. More patients in the renal function deterioration group presented with FeNa ≥1 % and RFI ≥1 %, and the uKIM-1 level was significantly higher than that of the stable renal function group (Table 5).

**Table 5:** Comparison between the renal function deterioration group and the stable renal function group after one-year follow up in patients with acute kidney injury

Characteristics	Stable renal function (*n* = 111)	Renal function deterioration (*n* = 73)	*P*
Age, years	42.00 (29.00, 59.00)	59.00 (45.00, 71.00)	0.000
Male sex	73 (65.77 %)	44 (60.27 %)	0.531
Baseline serum creatinine (mg/dl)	0.96 (0.76, 1.32)	2.17 (0.99, 3.22)	0.000
Baseline estimated GFR (ml/min/1.73 m^2^)	84.39 (50.57, 111.86)	31.21 (17.09, 61.04)	0.000
Baseline 24 hUTP (g/24 h)	3.38 (1.44, 8.83)	2.23 (1.48, 9.06)	0.802
Peak serum creatinine (mg/dl)	1.92 (1.27, 3.04)	3.43 (2.31, 4.63)	0.000
uKIM-1 (ng/mg)	1.57 (0.70, 3.89)	4.41 (1.69, 8.72)	0.000
FeNa ≥1 % (*n* (%))	61 (54.95 %)	65 (89.04 %)	0.000
RFI ≥1 % (*n* (%))	71 (63.96 %)	65 (89.04 %)	0.000

*GFR* glomerular filtration rate, *UTP* Urinary protein excretion, *uKIM-1* urinary kidney injury molecule-1, *FeNa* fractional excretion of sodium, *RFI* renal failure index

Gradual Cox regression analysis indicated that older patients had lower basic eGFR, higher uKIM-1, and poorer long-term renal prognosis. The risk of renal function deterioration after AKI increased by 3.3 % for each one-year increase in age. The risk of renal function deterioration after AKI increased by 2.4 % for each decrease of 1 ml/min/1.73 m^2^ of basic eGFR. The risk of renal function deterioration after AKI increased by 1.8 % for each 1 ng/mg increase in uKIM-1 (Table 6).

**Table 6:** Cox regression shows the independent risk factors for renal function progression in patients with acute kidney injury

	Univariate analysis		Multivariate analysis	
Characteristics	Relative risk	*P*	Relative risk	*P*
Age, years	1.033 (1.018, 1.047)	0.000	1.032 (1.008, 1.057)	0.009
Male sex				
Baseline estimated GFR (ml/min/1.73 m^2^)	0.976 (0.965, 0.987)	0.000	0.974 (0.962, 0.986)	0.000
Peak serum creatinine (mg/dl)	1.002 (1.001, 1.003)	0.001		
uKIM-1 (ng/mg)	1.018 (1.008, 1.029)	0.000	1.018 (1.004, 1.031)	0.009
FeNa ≥1 %	3.314 (1.622, 6.768)	0.001		
RFI ≥1 %	2.670 (1.199, 5.948)	0.016		

*GFR* glomerular filtration rate, *uKIM-1* urinary kidney injury molecule-1, *FeNa* fractional excretion of sodium, *RFI* renal failure index

**Fig. 8:**
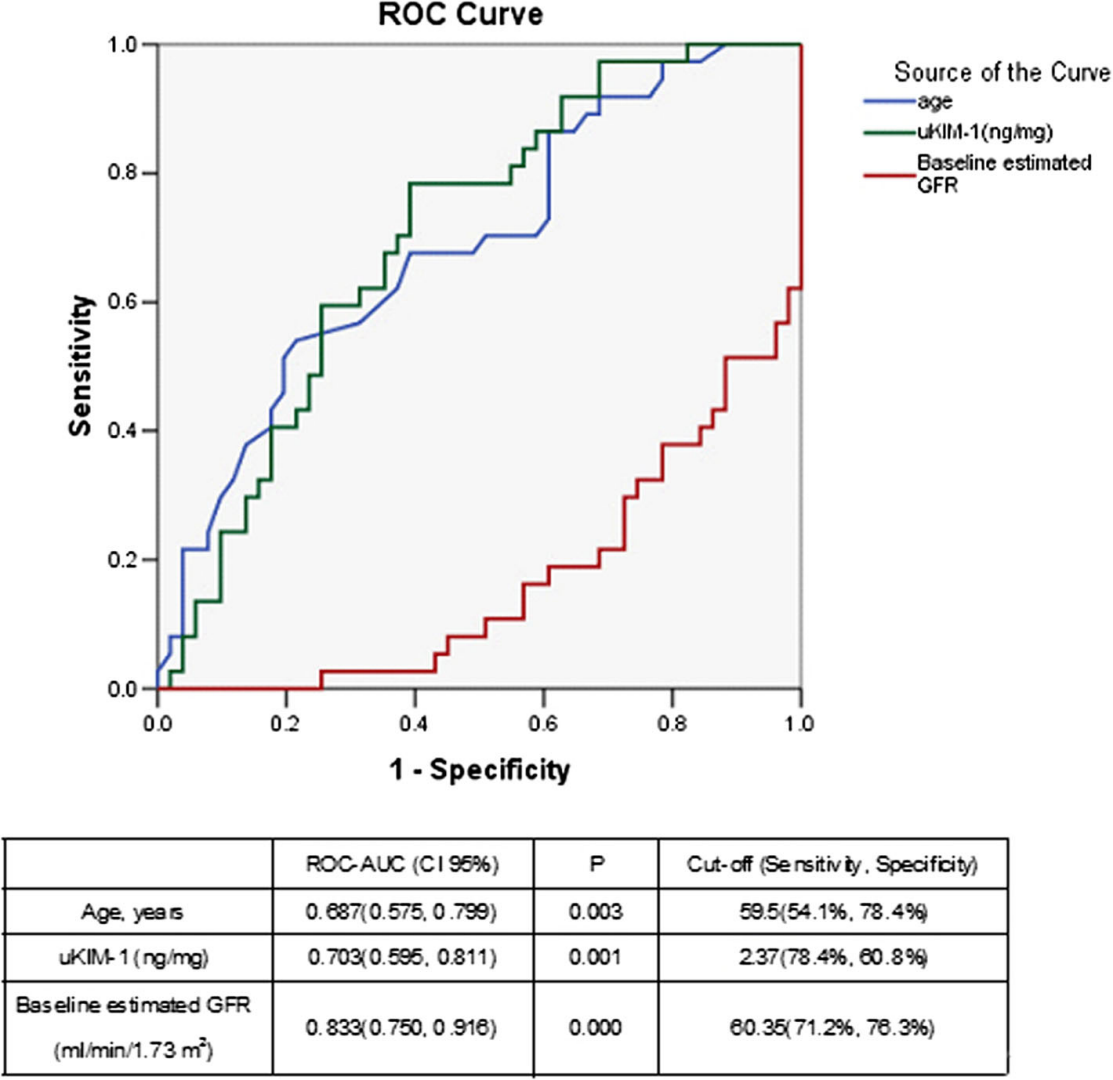
Receiver operating characteristic area under the curve (*ROC-AUC*) of renal function progression in patients with acute kidney injury. *uKIM-1* urinary kidney injury molecule-1, *GFR* glomerular filtration rate

The ROC-AUC values for the prediction of renal function deterioration with age, uKIM-1 level and basic eGFR level were 0.687, 0.703 and 0.833, respectively. When the age cutoff point was 59.5 years, the sensitivity was 54.1 %, and the specificity 78.4 %. When the cutoff point of the uKIM-1 level was 2.37 ng/mg, the sensitivity was 78.4 %, and the specificity 60.8 %. When the cutoff point of the eGFR level was 60.35 ml/min/1.73, the sensitivity and specificity were 71.2 % and 76.3 %, respectively (Fig. 8).

**Fig. 9:**
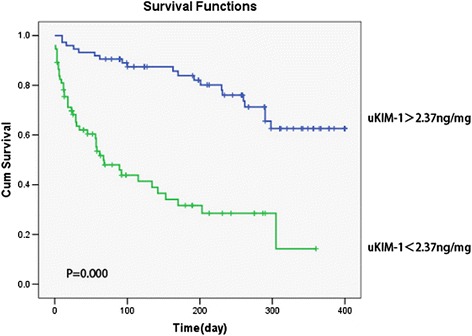
Urinary kidney injury molecule-1 (*uKIM-1*) and kidney prognosis in patients with acute kidney injury. uKIM-1 > 2.37 ng/mg was positively related with poor prognosis. *Cum* cumulative

The K-M curve revealed that uKIM-1 > 2.37 ng/mg positively correlated with poor long-term renal prognosis (*P* = 0.000) (Fig. 9).

## Discussion

The incidence rate of AKI is high, and is showing a trend of annual increase. AKI is common in hospitalized patients, especially critical patients; critical patients with AKI have high mortality rates. Recent epidemiological data have indicated that even mild and reversible AKI leads to persistent injuries of renal tissues, and severe AKI results in an irreversible decrease in renal function, which increases the risk of death. Several observational studies have demonstrated that acute renal injuries are significantly correlated with the subsequent development of chronic renal disease [13]. For example, many observational studies have demonstrated that some renal function is often recovered in a large proportion of patients with acute renal injury and patients without previous renal disease, but symptoms in these patients subsequently develop end-stage chronic renal disease. However, the long-term prognosis of AKI is not clear, and markers of early AKI prognosis are lacking.

This study followed 184 patients with AKI of different etiological types, and found that nearly 40 % of patients presented with deterioration in renal function after one year. On further analysis of these patients with AKI, 86 patients had recovered renal function within short-term follow up (within 48 h) and 98 patients did not recover renal function: 50 % of patients who did not recover renal function within 48 h had renal function deterioration within one year. Previous studies [14, 15] have also demonstrated that AKI results in CKD and affects the course of CKD. AKI is generally reversible when serum creatinine concentrations recover, but there may be subclinical injury of the kidney and other organs, resulting in CKD and ESRD.

Methods that indicate the prognosis of AKI during early stages are lacking. Basic studies [16, 17] have demonstrated that renal ischemia reperfusion injury alters the permeability of endothelial cells, decreases blood capillaries around the tubules, and creates an oxygen deficiency around the tubules and in the tissue space, which eventually results in fibrosis and gradually changes the kidney structure. Therefore, these patients develop CKD more easily. KIM-1 is an outstanding marker of renal injury [18], and KIM-1 is increased in prerenal AKI. KIM-1 increases significantly in renal AKI [19]. The fractional excretion of FeNa and the RFI are often used to define renal AKI, but there are certain limitations [20]. This study demonstrated that uKIM-1 was significantly increased in patients with renal AKI. The ROC-AUC indicated that high uKIM-1 was valuable in the diagnosis of renal AKI. Thus, high uKIM-1 indicates a high possibility of severe renal injury.

In previous studies of obstructive nephropathy [21, 22], uKIM-1 in patients with deterioration of renal function were significantly higher than in patients with stable renal function among patients with obstructive nephropathy who developed AKI. After one year, uKIM-1 in the patients with deterioration in renal function was significantly higher than that patients with stable renal function in 90 patients with obstructive nephropathy, regardless of their AKI status, which indicates that uKIM-1 has a predictive value in the long-term prognosis of patients with obstructive nephropathy. A large clinical study [23] performed renal puncture biopsy in patients with renal disease of different etiological types and demonstrated that KIM-1 is secreted in proximal renal tubular epithelial cells; further, the tissue expression of KIM-1 is associated with fibrosis and inflammation. uKIM-1 reflects KIM-1 levels in tissues, which is related to renal tubulointerstitial inflammation and renal function. This relationship indicates that KIM-1 is not only a biological marker of acute renal proximal tubular injury but also a marker of tubulointerstitial chronic inflammation and fibrosis [24].

This study demonstrated that uKIM-1 levels in the renal function deterioration group were significantly higher than those in the stable renal function group in patients with renal AKI after a regular one-year follow up. The risk of renal function deterioration following AKI increased by 6.4 % for each 1 ng/mg increase in uKIM-1. Analysis of the ROC curve indicates that the uKIM-1 level better predicted the long-term prognosis of patients with renal AKI. We further analyzed the 184 included patients with AKI, and the results indicated that uKIM-l in the renal function deterioration group was significantly higher than that in the stable renal function group. Age, basic eGFR, and uKIM-1 were risk factors for poor renal prognosis. The K-M curve demonstrated that uKIM-1 > 2.37 ng/mg positively correlated with poor long-term renal prognosis. Our study indicates that uKIM-1 better predicts the long-term prognosis of patients with AKI.

This was a single-center study with a small sample size; changes in renal function were followed up for one year, but no further follow up was performed to identify changes in marker levels. Therefore, a larger, multi-center study is required to verify the results of this study, and follow up must be performed to detect changes in marker levels during the progression of renal diseases, to further prove the value of uKIM-1 for the monitoring of renal prognosis.

## Conclusions

The prognosis of patients with AKI is poor, and there is a risk of renal function progression. High uKIM-1 indicates a high possibility of severe renal injury. uKIM-1 accurately predicted the renal prognosis of patients with AKI, and they may be used as an early screening indicator of poor renal prognosis. The results of this study suggest novel treatment strategies and may help clinical physicians to engage in early interventions to slow the progression of renal deterioration after AKI.

## Abbreviations

AKI: Acute kidney injury
CKD: Chronic kidney disease
eGFR: Estimated glomerular filtration rate
ESRD: End-stage renal disease
FeNa: Fractional excretion of sodium
RFI: Renal failure index
ROC-AUC: Receiver operating characteristic area under the curve
sCr: Serum creatinine
uCr: Urine creatinine
uKIM-1: Urinary kidney injury molecule-1
